# Transcriptome–Metabolome Analysis Reveals That Crossbreeding Improves Meat Quality in Hu Sheep and Their F_1_-Generation Sheep

**DOI:** 10.3390/foods14081384

**Published:** 2025-04-17

**Authors:** Liwa Zhang, Xuejiao An, Zhenfei Xu, Chune Niu, Zhiguang Geng, Jinxia Zhang, Haina Shi, Zhenghan Chen, Rui Zhang, Yaojing Yue

**Affiliations:** 1 Lanzhou Institute of Husbandry and Pharmaceutical Sciences, Chinese Academy of Agricultural Sciences, Lanzhou 730050, China; 17899319024@163.com (L.Z.); anxuejiao@caas.cn (X.A.); shihaina0716@163.com (H.S.); 19139107648@163.com (Z.C.); 2 Sheep Breeding Engineering Technology Research Center of Chinese Academy of Agricultural Sciences, Lanzhou 730050, China; 3 Key Laboratory of Animal Genetics and Breeding on Tibetan Plateau, Ministry of Agriculture and Rural Affairs, Lanzhou 730050, China; 4 Qingyang Research Institute of Agricultural Sciences, Qingyang 745000, China; 236xuzhenfei@163.com (Z.X.); gengzhiguang2@163.com (Z.G.); jinxia425@tom.com (J.Z.)

**Keywords:** crossbreed, *longissimus dorsi*, meat quality, transcriptomics, metabolomics

## Abstract

Consumers are increasingly demanding higher-quality mutton. Crossbreeding has been recognized as an effective means to improve meat quality. However, the phenomenon underlying these molecular system mechanisms remains largely unidentified. In this study, 48 male lambs aged 3 months were selected, including ♂ Hu sheep × ♀ Hu (HH, *n* = 16), ♂ Polled Dorset × ♀ Hu sheep F_1_ hybrid lambs (DH, *n* = 16), and ♂ Southdown × ♀ Hu sheep (SH, *n* = 16) F_1_ hybrid lambs, and raised in a single pen under the same nutritional and management conditions for 95 days. Then, seven sheep close to the average weight of the group were selected and fasted for 12 h prior to slaughter. By comparing the muscle fiber characteristics of the *Longissimus dorsi* of the three groups of sheep, and through transcriptomic and metabolomic analyses, we revealed molecular differences in the meat quality of Hu sheep crossbred with different parent breeds. The results of this study showed that muscle fiber diameter and cross-sectional area were significantly greater in the DH group than in the HH group, and collagen fiber content in the DH group was also significantly higher than in the HH group (*p* < 0.05). A total of 163 differential genes and 823 differential metabolites were identified in the three groups, most of which were related to muscle development and lipid metabolism. These included the AMPK signaling pathway, the PI3K-Akt signaling pathway, glycerophospholipid metabolism, and the related genes *EFHB*, *PER3*, and *PPARGC1A*. The results of this study offer valuable insights into the molecular mechanisms underlying the impact of crossbreeding on meat quality and provide a theoretical foundation for sheep crossbreed production.

## 1. Introduction

The demand for healthy meat products is increasing worldwide. China is the world’s largest market for future proteins [1]. China is also one of the largest consumers and producers of mutton in the world. Crossbreeding can improve meat quality and is widely used in sheep breeding. Hu sheep, an indigenous Chinese breed, exhibit advantageous traits including accelerated growth kinetics, enhanced reproductive efficiency, and non-seasonal estrous cycles [2]. Nevertheless, their carcass production efficiency, as measured by commercial dressing percentage and lean meat yield, remains suboptimal compared to specialized meat breeds. Southdown sheep are known for their gentle temperament, excellent meat quality, and excellent carcass traits. They have been introduced into many countries as sires for producing fat lambs [3]. Poll Dorset sheep are characterized by their rapid development and early maturity [4], which meet the current requirements for the slaughter age of sheep in China. Crossbreeding can generate heterosis and is an important means to improve production performance and meat quality. Texel and Segureña crossbreeding to produce F_1_-generation sheep demonstrated a higher average daily weight gain (ADG) and increased muscle protein content [5]. The F_1_-generation sheep of Mongolian crossed with Small-Tail Han sheep exhibited superiority in growth and development over purebred Small-Tail Han sheep [6]. In a comparison of Aldi and Damascus goats and their crossbred strains, crossbred goats showed higher heat tolerance. Compared to purebred Aardi goats, they exhibited superior production performance [7]. With the rapid advancement of high-throughput sequencing technology, transcriptomics and metabolomics have become widely employed in studying the quality traits of animal meat. RNA-Seq, particularly potent in this domain, furnishes compelling evidence for genetic regulatory mechanisms and is extensively utilized in meat quality research [8,9]. Metabolomic analysis offers insights into post-transcriptional regulation-induced metabolic changes, aiding in the study of metabolic characteristics and physiological variations. This approach is widely used to explore meat quality differences [10,11]. Omics-based association analysis integrates differences and complementarity between two omics studies to elucidate gene expression patterns and regulatory processes, widely applied in exploring meat quality mechanisms [12,13,14]. Our previous study demonstrated that crossing Hu sheep with Southdown and Polled Dorset sheep led to enhanced growth performance, higher slaughter rates, increased carcass weights, and greater net meat weights in the crossbred offspring [15]. Furthermore, the muscle protein content and amino acid levels in these hybrids were significantly higher than those in Hu sheep [16]. However, the regulatory mechanisms of crossbreeding for meat quality in sheep remain unclear. Therefore, this study aims to analyze the *longissimus dorsi* muscle of different sire crossbreeds through a combination of muscle fiber characteristics, transcriptomics, and metabolomics, with the objective of deepening our understanding of the complex molecular processes involved in sheep muscle fiber development and exploring the molecular mechanisms underlying the increased meat production in the F_1_ generation of crosses between Hu sheep and either Southdown or Poll Dorset sheep. The findings from the transcriptome and metabolome analyses reveal numerous differential genes and metabolites, which are crucial for the development of and genetic improvement in sheep germplasm resources.

## 2. Materials and Methods

### 2.1. Animals and Sample Collection

This experiment was carried out at Qingyang Huan County Sheep Breeding Co., Ltd., located in Qingyang, Gansu Province, China. The experimental design, procedures, and methods were previously published [15,16] and have been approved by the Lanzhou Institute of Husbandry and Pharmaceutical Sciences, Chinese Academy of Agricultural Sciences Institutional Animal Care and Use Committee, under the permit 2022-018. Briefly, a total of 48 male lambs aged 3 months were selected, including ♂ Hu sheep × ♀ Hu sheep (HH, *n* = 16), ♂ Polled Dorset × ♀ Hu sheep F_1_ hybrid lambs (DH, *n* = 16), and ♂ Southdown× ♀ Hu sheep F_1_ hybrid lambs (SH, *n* = 16), and raised in a single pen under the same nutritional and management conditions for 95 days (including a 15 day pre-test period). Lambs were fed a total mixed ration (TMR) mainly consisting of corn silage and grain mixtures to meet or exceed their nutritional requirements (Table S1). At the end of the experiment, seven sheep close to the average weight of the group were selected and fasted for 12 h prior to slaughter. About 1 cm × 2 cm × 0.5 cm *longissimus dorsi* muscle samples were collected from animals and fixed in 4% polyformaldehyde for muscle staining, and two additional 2 g *longissimus dorsi* muscle samples were stored in liquid nitrogen for RNA and metabolite extraction.

### 2.2. Muscle Staining

The fixed samples were dehydrated in a graded series of absolute ethanol solutions, embedded in paraffin, and sectioned. The sections were dewaxed and stained with hematoxylin and eosin (H&E). The sections were observed under an upright microscope (Nikon, ci-s, Tokyo, Japan). Slide Viewer 2.6 software (3DHISTECH, Hungary) was used to scan section images at 5× magnification to observe morphological features. ImageJ software (v1.8.0) was used to measure the diameter, area, and density of the muscle fibers.

For Masson staining, the paraffin sections were dewaxed and hydrated. They were then stained with Weigert’s iron hematoxylin for 5 min, rinsed briefly with running water, and then blued. After that, the sections were stained with ponceau acid fuchsin for 5 min and rinsed with running water. Subsequently, they were treated with phosphotungstic acid solution for approximately 5 min. After the excess liquid was removed, the sections were counterstained with aniline blue for 3 min. The sections were then rinsed with 1% acetic acid in water until no blue color was released from the sections. They were then rinsed briefly with 95% alcohol, dehydrated with absolute ethanol and a graded series of xylene, and finally mounted with neutral resin. The sections were observed under an upright microscope (Nikon, ci-s, Japan). Slide Viewer 2.6 software (3DHISTECH, Hungary) was used to scan section images at 5× magnification to observe morphological features. ImageJ software (v1.8.0) was used to measure the relative collagen content.

For fast and slow muscle immunofluorescence staining, after the tissue samples were processed according to the paraffin embedding method, the sections were dewaxed and subjected to antigen retrieval. A serum blocking solution was then applied for 1 h. The primary antibody was used to cover the section samples, incubated overnight at 4 °C. Subsequently, a secondary antibody labeled with HRP (horseradish peroxidase) was added. The antigen retrieval process was repeated, followed by a second staining with a different antibody, following the same steps as the first antibody staining. Finally, DAPI staining and mounting were performed, and the slides were stored in the dark. The prepared slides were observed and photographed under an inverted fluorescence microscope. Images were scanned at 5× magnification using Slide Viewer 2.6 software (3DHISTECH, Hungary). Four fields of view were selected from each slide, and the fluorescent areas of fast and slow muscle fibers were measured using ImageJ (v1.8.0) software.

### 2.3. Transcriptome Sequencing and Bioinformatics Analysis

Total RNA was extracted from *longissimus dorsi* tissue using the TransZol kit (TransGen Biotech, Beijing, China). The extracted total RNA was detected and quantified using a NanoPhotometer and Qsep400 bioanalyzer. Ribosomal RNA was removed from the total RNA to obtain mRNA. Fragmentation buffer was then added to fragment the RNA. First-strand cDNA was synthesized utilizing the M-MuLV reverse transcriptase system. Following this, with dNTPs serving as the substrate, the second strand of cDNA was synthesized in the presence of DNA polymerase I. The purified double-stranded cDNA underwent end repair, A-tailing, and the ligation of sequencing adapters. AMPure XP beads were used to select cDNAs around 200 bp in length. PCR amplification was performed, and the PCR products were purified again using AMPure XP beads to obtain the final library. After passing quality control, the library was sequenced using the Illumina platform (Beijing, China). The raw data were filtered using fastp (v0.23.2) to obtain clean reads. The clean reads were then aligned to the reference genome using HISAT2(2.2.1). The gene alignment was counted using featureCounts (v2.0.3), and the FPKM (Fragments Per Kilobase of transcript per Million mapped reads) for each gene was calculated based on gene length. Differential expression analysis was performed using DESeq2(1.22.1), with the screening criteria for differentially expressed genes (DEGs) being |log_2_Fold Change| ≥ 1 and FDR < 0.05. The annotated functional genes were subjected to Gene Ontology (GO) functional enrichment and Kyoto Encyclopedia of Genes and Genomes (KEGG) pathway enrichment analysis using the DAVID (v6.8) database and software. Additionally, the NCBI database was used to further investigate the functions of these entries and pathways, allowing for the selection of trait-related functional genes and pathways.

### 2.4. Metabolome Sequencing and Bioinformatics Analysis

First, accurately weigh 20 mg (±1 mg) of sample and place it in a centrifuge tube, and add 400 μL of 70% methanol aqueous internal standard extraction solution. Vortex the sample at 2500 r/min for 5 min and let it sit on ice for 15 min. Centrifuge at 12,000 r/min for 10 min at 4 °C. Pipette 300 μL of the supernatant into another centrifuge tube with the corresponding number and let it sit in a −20 °C freezer for 30 min. Centrifuge again at 12,000 r/min for 3 min at 4 °C. Pipette 200 μL of the supernatant into the corresponding vial liner for analysis. The chromatographic conditions are as follows: the chromatographic column is Waters ACQUITY UPLC HSS T3 C18 1.8 µm, 2.1 mm × 100 mm; mobile phase A is ultrapure water (0.1% formic acid); and mobile phase B is acetonitrile (0.1% formic acid). The elution gradient is set as follows: 0 min, water/acetonitrile (95:5 V/V), 11 min, 10:90 V/V, 12 min, 10:90 V/V, 12.1 min, 95:5 V/V, and 14.0 min, 95:5 V/V; the flow rate is 0.4 mL/min; the column temperature is 40 °C; and the injection volume is 2 μL. 

The acquisition conditions for mass spectrometry are detailed as follows: The electrospray ionization (ESI) source is maintained at a temperature of 500 °C, with the mass spectrometry voltage configured at 5500 V for positive ions and −4500 V for negative ions. The ion source gas I (GS I) is adjusted to 55 psi, gas II (GS II) to 60 psi, and curtain gas (CUR) to 25 psi. The collision-activated dissociation (CAD) parameter is set to its highest level. In the triple quadrupole (QTRAP) mass spectrometer, each ion pair undergoes scanning and detection based on the optimized declustering potential (DP) and collision energy (CE). The mass spectrometry data were processed using Analyst (v1.6.3), and metabolite characterization was based on the MWDB (metware database). The overall distribution among samples and the stability of the entire analytical process were observed. Orthogonal partial least squares discriminant analysis (OPLS-DA) was utilized to distinguish differences in metabolites among groups. Significant differences in metabolites between the two groups were screened using variable importance in projection (VIP ≥ 1) values and the *p*-value (*p* < 0.05) as thresholds for selecting differential metabolites (DMs). The KEGG database was utilized to perform metabolic pathway enrichment analysis on the DMs. *p* < 0.05 indicated significant enrichment in the KEGG pathways.

### 2.5. Comprehensive Analysis of Transcriptome and Metabolome Data

To uncover the regulatory mechanisms between gene expression and metabolites, we employed LC-MS/MS technology to perform integrated biological annotation and correlation analysis on transcriptome and metabolome data. Pearson correlation analysis was utilized to assess the associations between differentially expressed genes and differentially abundant metabolites, and further insights into their biological functions were revealed based on KEGG pathway enrichment statistical analysis.

### 2.6. Quantitative Real-Time PCR (qRT-PCR) Validation of DEGs

Total RNA was extracted using RNA extractor (Xavier Biotechnology Co., Ltd., Wuhan, China), and first-strand cDNA synthesis was performed using a reverse transcriptase kit (Xavier Biotechnology Co., Ltd., Wuhan, China). qRT-PCR was performed on a fluorescence quantitative PCR instrument (Bio-rad), with a reaction program of pre-denaturation at 95 °C for 30 s, 95 °C for 15 s, 60 °C for 30 s, 72 °C for 10 s, 40 cycles, 65–95 °C (fluorescence signals were collected once for every 0.5 °C increase in temperature). Primer information is shown in Table S2, with the GAPDH gene as the internal reference gene. Three replicates were performed independently for each gene in the qRT-PCR reaction. The relative expression of each gene was calculated by the 2^−ΔΔCT^ method.

### 2.7. Statistical Analysis

All data were checked for normality and outliers using SPSS version 26.0 (IBM Corp., Armonk, NY, USA) before any statistical analyses were conducted. One-way ANOVA and Tukey’s post hoc test were used to analyze the data of muscle fiber characteristics and relative gene expression. Data are presented as mean ± standard deviation (SD). DEGs and DMs were selected between the two groups. Pearson correlation analysis was utilized to assess the associations between differentially expressed genes and differential metabolites (r > 0.8, *p* < 0.05). GraphPad Prism version 8.0 (GraphPad Software, La Jolla, CA, USA) was used to draw the statistical maps. Seven biological replicates were produced in all analyses.

## 3. Results

### 3.1. Determination of Muscle Fiber Characteristics of Hu Sheep and Their Crossbred F_1_-Generation Sheep

**Figure 1:**
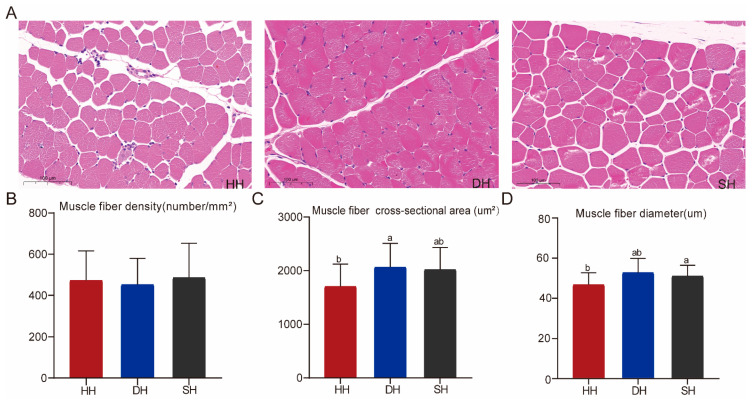
Muscle fiber morphology traits in the HH, DH, and SH groups. (**A**) Tissue sections of the *longissimus dorsi* muscle were stained with HE (5× magnification). (**B**–**D**) Comparison of muscle fiber density, muscle fiber cross-sectional area, and muscle fiber diameter among the three groups. All data are expressed as mean ± SD (*n* = 7). Different letters indicate significant differences (*p* < 0.05). HH: ♂ Hu × ♀ Hu sheep; DH: ♂ Polled Dorset × ♀ Hu sheep; SH: ♂ Southdown × ♀ Hu sheep.

The results of HE staining show that cell nuclei appeared blue, and the muscle fibers appeared red (Figure 1A). The muscle fiber density was not significantly different among the three groups (*p* > 0.05, Figure 1B). The muscle fiber diameter and cross-sectional area in the DH group were significantly higher than those in the HH group (*p* < 0.05, Figure 1C,D).

**Figure 2:**
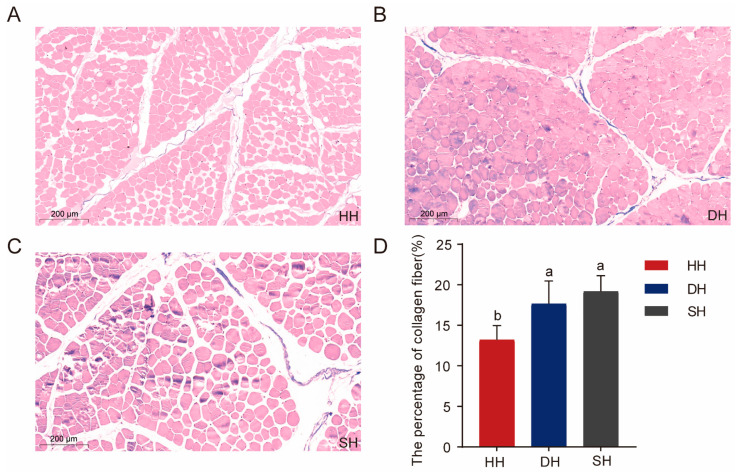
Collagen fiber content in the HH, DH, and SH groups using Masson staining. (**A**–**C**) Tissue sections of *dorsal longissimus* muscle were stained with Masson (5× magnification). (**D**) Comparison of collagen fiber content among the three groups. All data are expressed as mean ± SD (*n* = 7). Different letters indicate significant differences (*p* < 0.05). HH: ♂ Hu × ♀ Hu sheep; DH: ♂ Polled Dorset × ♀ Hu sheep; SH: ♂ Southdown × ♀ Hu sheep.

Masson staining was used to determine the collagen fiber content of crossbred F_1_-generation sheep. Collagen fibers, mucus, and cartilage were stained blue, muscle fibers, cellulose, and red blood cells appeared red, and the cell nuclei were blue-black (Figure 2A–C). Similarly to the results for muscle fiber diameter and cross-sectional area, the collagen fiber content in the DH and SH groups was significantly higher than that in the HH group, increasing by 4.43% and 5.97%, respectively (*p* < 0.05, Figure 2D).

**Figure 3:**
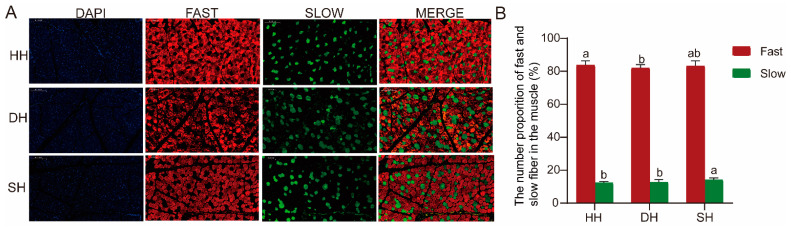
Muscle fiber type composition in the HH, DH, and SH groups. (**A**) Immunofluorescence staining detected the distributions of fast and slow muscle fibers. (**B**) The numerical proportion of fast and slow fibers in the muscle. All data are expressed as mean ± SD (*n* = 7). Different letters indicate significant differences (*p* < 0.05). HH: ♂ Hu × ♀ Hu sheep; DH: ♂ Polled Dorset × ♀ Hu sheep; SH: ♂ Southdown × ♀ Hu sheep.

The immunofluorescence staining method was employed to detect the distribution of fast and slow muscle fibers among the three groups. Fast muscle fibers stained red, while slow muscle fibers stained green (Figure 3A). The proportions of slow and fast muscle fibers in the three groups are shown in Figure 3B; in the comparative analysis of the three groups, the proportion of slow muscle fibers in the SH group was significantly higher than that in the other two groups, and the proportion of fast muscle fibers was significantly higher in the HH group than in the DH group (*p* < 0.05).

### 3.2. Transcriptome Profiling of Muscle

**Figure 4:**
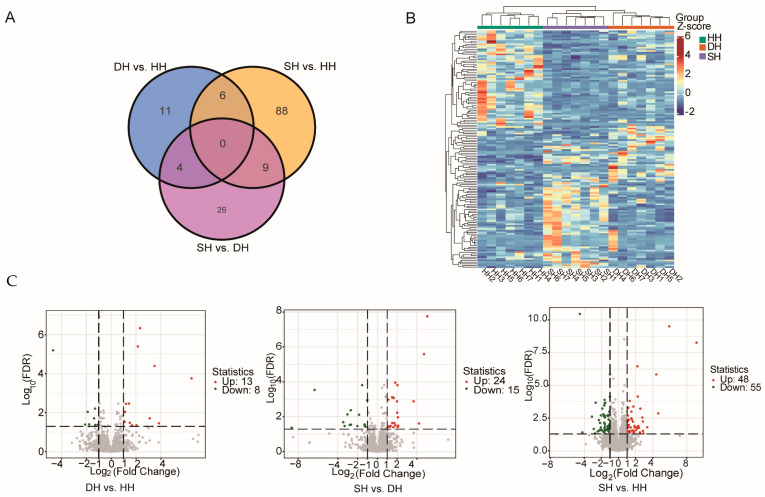
Transcriptomic comparisons of the *longissimus dorsi* for the DH vs. HH, SH vs. DH, and SH vs. HH groups. (**A**) Venn plot of three groups. (**B**) Heatmap of differential gene clustering. (**C**) Volcano plots of the DEGs. Red dots represent up-regulated DEGs; green dots represent down-regulated DEGs.

After the filtering and quality control of the raw data, an average of 303,803,336 high-quality clean reads were obtained from the three groups. The Q30 base distribution ranged from 94.10% to 94.39%, with an average GC content of 51.76%. The overall sequencing error rate was 0.03%, and over 91% of the reads in each sample could be aligned to the sheep reference genome (Table S3). These results indicate that the overall quality of the sequencing data is suitable for subsequent in-depth analysis. Based on the transcriptome sequencing results, DEGs were screened using the criteria of |log2Fold Change| ≥ 1 and FDR < 0.05 (Table S4). A total of 144 DEGs were identified in the three groups of F_1_-generation sheep, among which DH vs. HH shared 4 DEGs with the SH vs. DH group and 6 DEGs with the SH vs. HH group. SH vs. DH shared nine DEGs with SH vs. HH (Figure 4A). The heatmap of the DEGs demonstrates good clustering among the three groups of samples (Figure 4B). According to the volcanic map of DEGs (Figure 4C), a total of 21 DEGs were found in group DH vs. HH, of which 13 genes were up-regulated and 8 genes were down-regulated. There were 39 genes in the SH vs. DH group, including 24 up-regulated genes and 15 down-regulated genes. In the SH vs. HH group, 103 DEGs were identified, of which 48 genes were up-regulated and 55 genes were down-regulated.

**Figure 5:**
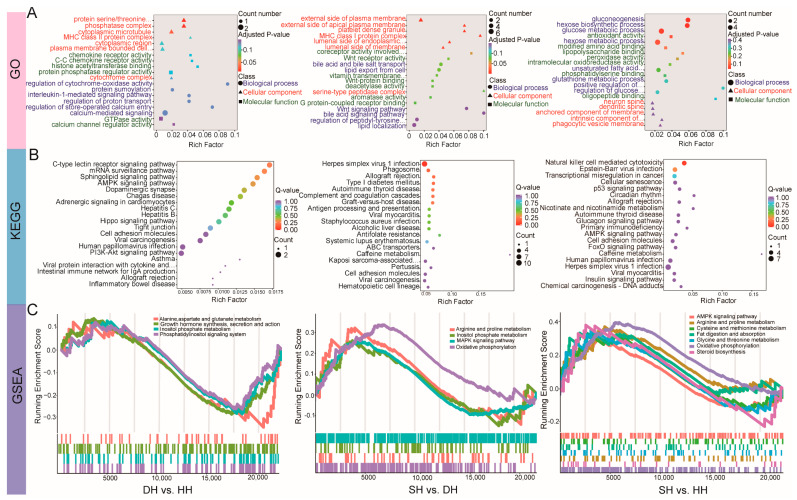
Functional enrichment analysis of the *longissimus dorsi* samples of the DH vs. HH, SH vs. DH, and SH vs. HH groups. (**A**) DEG GO enrichment histogram. The horizontal axis indicates the number of differential genes annotated to the entry, and the vertical axis indicates the name of the GO entry. The numbers in the figure indicate the number of differential genes annotated to the entry, the ratio of the number of differential genes annotated to the GO entry to the total number of differential genes is shown in parentheses, and the label on the far right represents the classification to which the GO entry belongs. (**B**) KEGG enrichment scatter plot of DEGs. The horizontal axis represents the Rich factor; the larger the Rich factor, the greater the degree of enrichment. The vertical axis represents the KEGG pathway; the larger the point, the greater the number of differential genes enriched in the pathway; the redder the color of the point, the more significant the enrichment. (**C**) Gene set enrichment analysis of the DH vs. HH, SH vs. DH, and SH vs. HH groups. The path name in the figure has been abbreviated, please refer to the complete name of the Supplementary Materials.

To further understand the functions of DEGs, we conducted GO and KEGG functional enrichment analyses (Table S5). The GO functional analysis revealed that the DEGs in the DH vs. HH group were significantly enriched (*p* < 0.05) in 99 GO terms (Figure 5A), including 65 terms related to biological process (BP), 18 related to cellular component (CC), and 16 related to molecular function (MF). The DEGs were primarily enriched in calcium-mediated signaling (GO:0019722), calcium channel regulator activity (GO:0005246), protein phosphatase regulator activity (GO:0019888), and protein serine/threonine phosphatase complex (GO:0008287). The DEGs in the SH vs. DH group were significantly enriched (*p* < 0.05) in 280 GO terms (Figure 5A), including 178 terms related to BP, 35 related to CC, and 67 related to MF. Among these, the DEGs were primarily enriched in lipid export from cell (GO:0140353), bile acid and bile salt transport (GO:0015721), icosanoid secretion (GO:0032309), and bile acid signaling pathway (GO:0038183). The DEGs in the SH vs. HH group were significantly enriched (*p* < 0.05) in 261 GO terms (Figure 5A), including 214 terms related to BP terms, 16 related to CC terms, and 31 related to MF terms. Among these, the DEGs were primarily enriched in gluconeogenesis (GO:0006094), hexose biosynthetic process (GO:0019319), glucose metabolic process (GO:0006006), and hexose metabolic process (GO:0019318). KEGG enrichment analysis revealed (Table S6) that a total of 15 signaling pathways were significantly enriched (*p* < 0.05) in the DH vs. HH group (Figure 5B), with the DEGs primarily enriched in the AMPK signaling pathway and PI3K-Akt signaling pathway. In the SH vs. DH group, 26 pathways were significantly enriched (*p* < 0.05, Figure 5B), with the DEGs mainly enriched in the herpes simplex virus 1 infection and phagosome pathways. For the SH vs. HH group, 25 pathways were significantly enriched (*p* < 0.05, Figure 5B), with the DEGs predominantly enriched in the herpes simplex virus 1 infection, Epstein–Barr virus infection, natural killer cell-mediated cytotoxicity, transcriptional misregulation in cancer, and human papillomavirus infection pathways. Genome enrichment analysis (GSEA) (Figure 5C) revealed that for DH vs. HH, the DEGs are enriched in alanine, aspartate and glutamate metabolism, growth hormone synthesis, secretion and action, inositol phosphate metabolism, and phosphatidylinositol signaling systems, and the expression of these pathways trends in the same direction. For SH vs. DH, the DEGs are enriched in arginine and proline metabolism, inositol phosphate metabolism, the MAPK signaling pathway, and oxidative phosphorylation, and the expression of these pathways trends in the same direction. For SH vs. HH, the DEGs are enriched in the AMPK signaling pathway, arginine and proline metabolism, cysteine and methionine metabolism, fat digestion and absorption, glycine, serine and threonine metabolism, oxidative phosphorylation, and steroid biosynthesis, and the enrichment trends of these pathways are generally consistent.

### 3.3. LC–MS Metabolomic Analysis in Muscle

**Figure 6:**
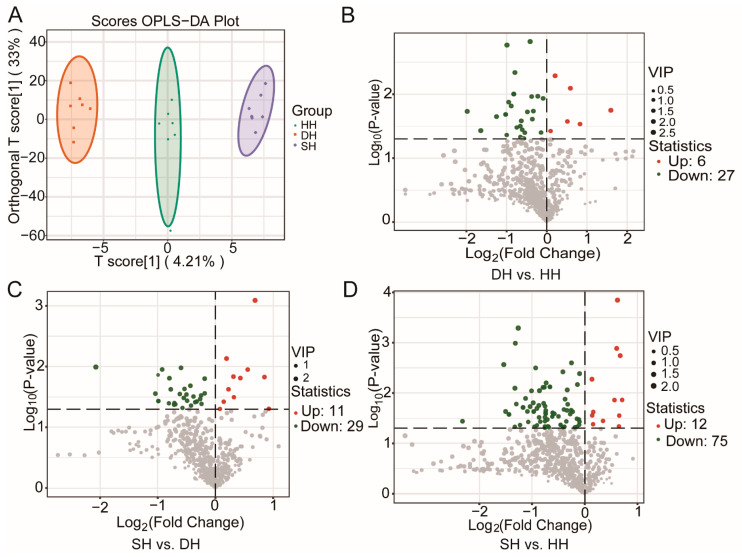
LC-MS/MS analysis of *longissimus dorsi* metabolic profiles for the DH vs. HH, SH vs. DH, and SH vs. HH groups. (**A**) OPLS-DA Score Chart. (**B**–**D**) DM volcano map.

Through the use of the LC-MS/MS method to compare the differences in muscle metabolites between Hu sheep and their crossbred F_1_-generation sheep, a total of 823 metabolites were detected across the three groups (Table S7). The primary metabolites included amino acid and its metabolites (25.39%), organic acid and its derivatives (13.49%), GP (9.84%), nucleotide and its metabolites (9.48%), benzene and substituted derivatives (7.90%), heterocyclic compounds (6.56%), and alcohol and amines (5.71%). Through the analysis of metabolomic data using the OPLS-DA model, significant differences were identified among the metabolic profiles of the three meat quality groups (Figure 6A). With VIP ≥ 1 and *p* < 0.05 as screening criteria, the DMs identified for the three groups are shown in Table S8. A total of 33 DMs were identified for the DH vs. HH combination, of which 6 DMs were up-regulated and 27 DMs were down-regulated (Figure 6B). For the SH vs. DH combination, 40 DMs were identified, of which 11 were up-regulated and 29 DMs were down-regulated (Figure 6C). For SH vs. DH, 87 DMs were identified, of which 12 DMs were up-regulated and 75 DMs were down-regulated (Figure 6D).

**Figure 7:**
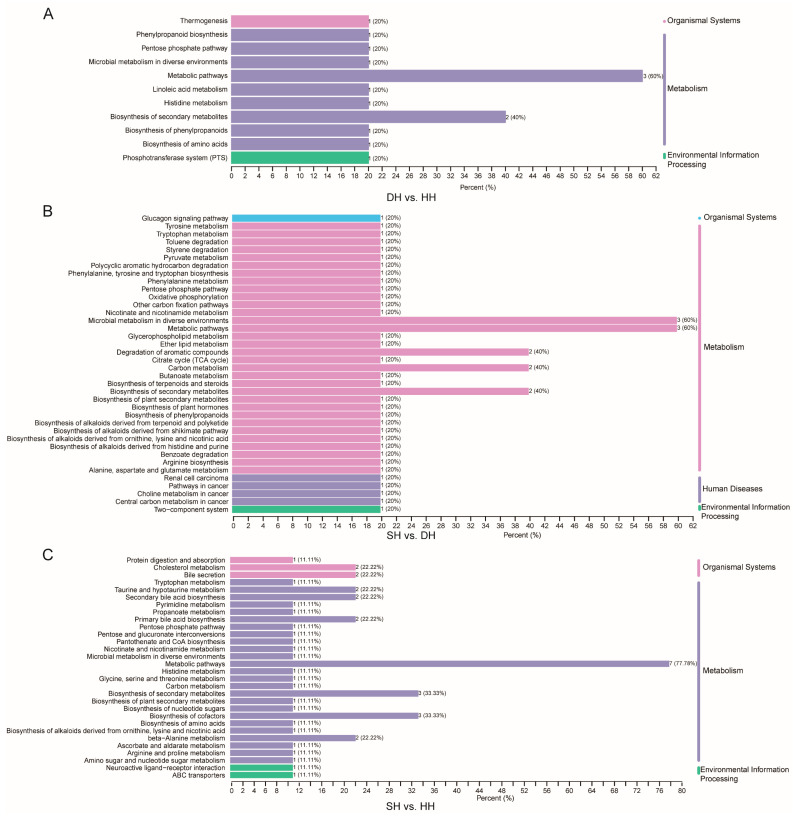
DM KEGG classification map of the DH vs. HH, SH vs. DH, and SH vs. HH groups. The vertical axis is the name of the KEGG metabolic pathway, and the horizontal axis is the number of DMs annotated to the pathway and the ratio of that number to the total number of differential metabolites for which they were annotated. (**A**–**C**) KEGG enrichment bar chart of the DH vs. HH, SH vs. DH, and SH vs. HH groups.

Based on KEGG pathway enrichment analysis (Table S9), DH vs. HH had nine DMs enriched in a total of 12 pathways (*p* < 0.05), with the majority of the DMs enriched in metabolic pathways and thermogenesis (Figure 7A). SH vs. DH had 16 DMs enriched in a total of 43 pathways (*p* < 0.05). Most of these DMs were enriched in metabolic pathways, tyrosine metabolism, carbon metabolism, butanoate metabolism, and nicotinate and nicotinamide metabolism (Figure 7B). SH vs. DH had 28 DMs enriched in a total of 68 pathways (*p* < 0.05), with most of the differential metabolites enriched in beta-alanine metabolism, purine metabolism, metabolic pathways, and nucleotide metabolism, biosynthesis of cofactors, thermogenesis, ABC transporters, taurine and hypotaurine metabolism, and biosynthesis of amino acids (Figure 7C).

### 3.4. Transcriptome and Metabolome Integration Analysis

**Figure 8:**
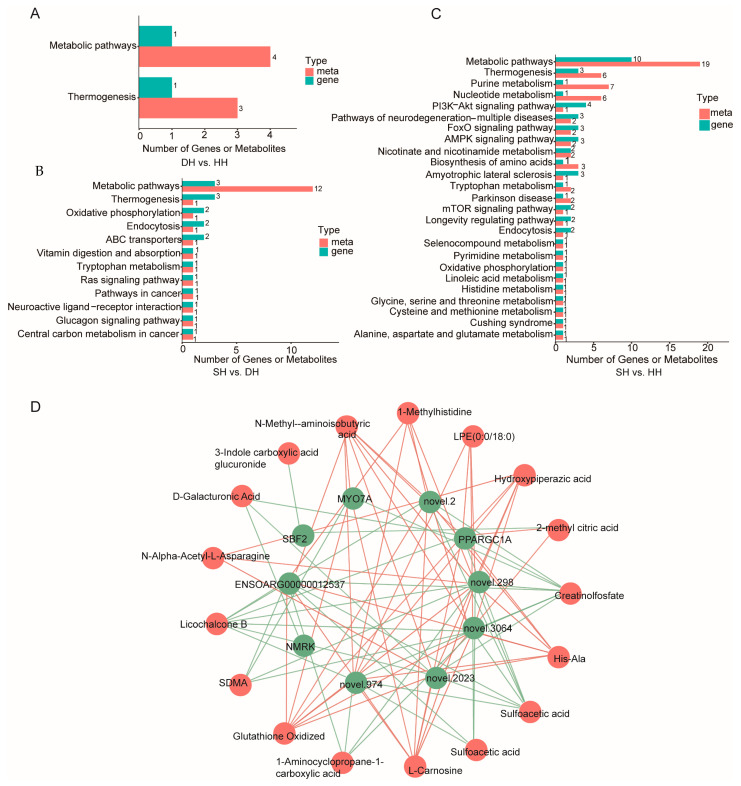
Comprehensive analysis of transcriptomics and metabolomics. (**A**–**C**) KEGG enrichment analysis bar graph: horizontal axes represent the number of differential metabolites and DEGs enriched in the pathway, vertical axes represent KEGG pathway names, and red and green bars represent the metabolome and transcriptome, respectively. (**D**) Correlation network diagram: a green circle is the name of the DEG, a red circle is the name of the DM, the thickness of a line represents the level of correlation, a green line represents negative correlation, and a red line represents positive correlation (*p* < 0.05).

The comprehensive KEGG analysis conducted on the two omics datasets is presented in Table S10, revealing that the DH vs. HH comparison shared enrichment in two pathways. Among these, thermogenesis was significantly enriched (*p* < 0.01), encompassing one DM compound and four DEGs (Figure 8A). The SH vs. DH comparison was jointly enriched in 12 pathways, with ABC transporters (one metabolite and two genes) and thermogenesis (one metabolite and three genes) being significantly enriched (*p* < 0.05, Figure 8B). For the SH vs. HH comparison, a total of 26 pathways were jointly enriched, with nicotinate and nicotinamide metabolism (two metabolites and two genes), AMPK signaling pathway (two metabolites and three genes), FoxO signaling pathway (two metabolites and three genes), and purine metabolism (seven metabolites and three genes) being significantly enriched (*p* < 0.05, Figure 8C). Selected DEGs and DMs, along with their calculated correlation results, are presented in Table S10. The results indicated that the DM 1,3-dicyclohexylurea in the DH vs. HH group showed a significant positive correlation with GEM (*p* < 0.05) and significant negative correlations with *EFHB* and *FAM124B* (*p* < 0.05). O-methoxybenzoic acid exhibited a significant negative correlation with *EFHB* (*p* < 0.05) and a significant positive correlation with *TEKT2* (*p* < 0.05, Table S10). Based on the correlation analysis of DEGs and DMs across the three groups, highly correlated DEGs and DMs for the two sample groups were plotted in a correlation network (Figure 8D), and we found *PPARGC1A* to be associated with hydroxypiperazine and His-Ala.

### 3.5. Detection of DEG Expression in Longissimus Dorsi by qRT-PCR

**Figure 9:**
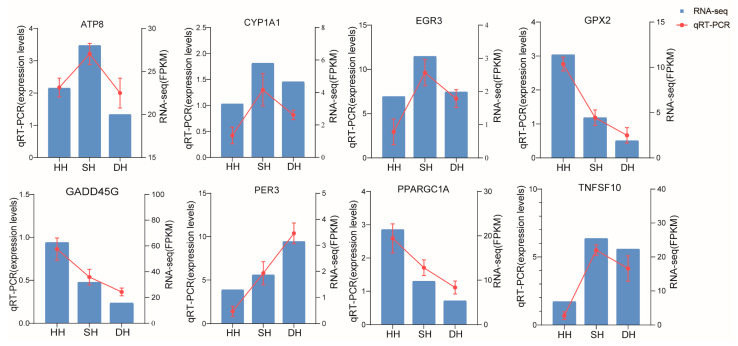
Validation of RNA-Seq by qRT-PCR analysis.

To validate the transcriptomic data, we randomly selected eight DEGs for the assessment of mRNA levels using qRT-PCR. As shown in Figure 9, the qRT-PCR results show that the expression patterns of the genes are consistent with the RNA-seq data, indicating that the transcriptomic results are reliable.

## 4. Discussion

Meat quality, as a critical economic trait for measuring the production performance of sheep, influences consumers’ purchasing power and satisfaction. Given that mutton is a high-quality source of protein for humans [17], this study explored the differences in meat quality among hybrid offspring based on muscle fiber indices and collagen fiber indices. Myofibers comprise the basic structure of muscle, and their number and size determine muscle quality and growth potential [18]. Improved meat tenderness and enhanced sensory quality are positively correlated with reduced muscle fiber diameter and increased fiber density, as these structural characteristics directly influence textural properties during mastication [19,20]. Muscles in the DH group exhibited a significantly greater fiber diameter and cross-sectional area compared to the HH group (*p* < 0.05), while fiber density showed no significant intergroup difference, suggesting superior tenderness characteristics in HH-group muscles. Muscle tenderness depends on various factors, including intramuscular fat and moisture content, as well as muscle fiber structure. Almost all muscle fibers are formed before birth, and the later stages of growth are mainly characterized by the hypertrophy and type transformation of muscle fibers [21]. The growth and development of muscle in livestock is a complex multi-stage process, regulated by many factors, and requires further investigation in conjunction with other meat quality indicators.

Fibrillar collagen predominates within the subendothelial matrix, serving as a significant constituent that actively participates in the composition of this layer [22]; it is also the main connective tissue protein in the animal body, accounting for 20% to 25% of the body’s total protein [23]. It has been shown that collagen content and collagen cross-linking play an important role in influencing meat tenderness. Collagen fiber content is negatively correlated with meat tenderness, while heat-soluble collagen fiber content is positively correlated with it [24]. In this experiment, the total collagen fiber content of the HH group was significantly greater than that of the DH group, which indicates that this may be one of the reasons for the difference in meat quality between the two groups.

The composition of different functional muscle fibers in skeletal muscle has an impact on some muscle-related diseases and meat quality. Slow-twitch muscle fibers demonstrate strong resistance to fasting, fatigue, diabetes, and atrophy [25,26]. Furthermore, the proportion of slow-twitch muscle fibers in skeletal muscle is positively correlated with the meat quality of livestock and poultry [27]. In this study, the proportion of slow-twitch muscle fibers in the SH group was significantly higher than that in the other two groups. This could be one of the reasons for the differences in meat quality among the three groups, suggesting to some extent that the meat quality of the hybrid offspring has been improved.

The analysis of DEGs among the three groups revealed that the majority of them are involved in the AMPK signaling pathway, PI3K/Akt signaling pathway, and metabolic pathways. The DEGs between the DH vs. HH groups are primarily enriched in calcium-mediated signaling, AMPK signaling pathway, PI3K/Akt signaling pathway, tight junction, and sphingolipid signaling pathway. The DEGs between the SH and DH groups are mainly enriched in the MHC protein complex, allograft rejection, and antigen processing and presentation, as well as antigen processing and presentation of peptide antigen. The SH vs. HH group primarily shows enrichment in reverse transcription, p53 signaling pathway, nicotinate and nicotinamide metabolism, cell adhesion molecules, and FoxO signaling pathway. The CaMKII family is a multifunctional protein kinase with serine/threonine specificity, which regulates various cellular activities through calcium ions [28]. Elevated Ca^2+^ was found to lead to the increased phosphorylation of CAMKII and p38MAPK in PPARγ pigs, which affects myofibril type and oxidative capacity [29]. The AMPK and PI3K/AKT signaling pathways play key roles in skeletal muscle growth and development and fatty acid metabolism [30,31]. When muscle cells are injured, the AMPK downstream molecule phosphoinositide 3-kinase (PI3K) Ser/Thr protein kinase B (serine/threonine protein kinase B, PKB, i.e., AKT) activates mTOR, resulting in myofiber hypertrophy [32]. The results of this study indicate that muscle cell repair and muscle fiber development are relatively better in the DH group.

The *PER3* gene belongs to the Period gene family and was cloned in 1998. Its protein sequence shares approximately 37% homology with *PER1* and *PER2*, which are also members of the same gene family [33,34]. Studies on gene *PER3* have focused on animal circadian rhythms, reproduction, gut health, and cancer [35,36,37]. However, other studies have found that *PER3* is related to fat metabolism. Zhang et al. studied the efficacy of bitter ginseng soup in treating hyperlipidemic rats and found that bitter ginseng soup reduced blood lipid levels in rats by modulating metabolic and circadian pathways [38]. Martín-Reyes F et al. [39] found that waist circumference was negatively correlated with peak *PER3* in visceral and subcutaneous fat studies in patients with obesity. Bakhtiarizadeh et al. [40] found that genes such as *ACSL1*, *FASN*, *PER3*, and *BMP6* were associated with caudal fat deposition in sheep by transcriptome sequencing. This study found that PER3 is mainly up-regulated in pathways such as the entrainment of circadian clock by photoperiod, photoperiodism, and entrainment of circadian clock in the SH vs. HH group. It is speculated that PER3 may cause differences in fat metabolism by regulating the circadian rhythm of sheep, which is one of the reasons for the difference in meat quality between the SH vs.HH groups. *EFHB* is a cytosolic protein composed of 833 amino acids with two EF-hand domains. As a member of the EF-hand domain family, *EFHB* participates in regulating the interaction between STIM1 and SARAF [41]. Currently, studies on *EFHB* are more focused on human medicine including familial genetic diseases, oncology, and reproductive medicine. Some studies have also found that *EFHB* is related to mammalian skeletal muscle development. Cui et al. [42] conducted transcriptome sequencing to investigate the genes and specific mechanisms regulating muscle fiber formation in Tan sheep. They found differentially expressed genes such as *EFHB*, *FBXL5*, *ACACB*, and *ASAH1*. In the present study, transcriptome sequencing revealed that EFHB was mainly enriched in the axonemal microtubule and store-operated calcium entry. The peroxisome proliferator-activated receptor gamma coactivator 1-alpha (*PPARGC1A*) gene, also known as PGC-1α, is a crucial transcriptional activator that is widely expressed in various tissues and organs of animals and participates in multiple biological processes [43,44]. Ma et al. [45] found that *PPARGC1A* promotes mitochondrial biogenesis, skeletal muscle development, and intramuscular fatty acid oxidation. It was found that *PPARGC1A* could be a candidate gene for influencing pork quality traits, and the c.-2894G > A polymorphism in the 5′ upstream region of the *PPARGC1A* gene could be used as an effective genetic marker for improving pork quality [46,47]. In this study, the *PPARGC1A* gene was down-regulated in the SH vs. HH group, and was mainly enriched in the AMPK signaling pathway, glucagon signaling pathway, and insulin signaling pathway. The results were different from those of Kong et al. [48] and Zhang et al. [49], which might be due to the biological differences between different samples or different analyzing methods, which led to some differences in the experimental results, the specific reasons for which need to be further investigated. In addition, this study found that DEGs in the three groups were also enriched in cancer and certain disease pathways (*TNFSF10*, *ACKR4*, *FLT3*, *SIGLEC1*, etc.). A related study reported that adipocytes are major stromal cells that play a dynamic role in the cancer microenvironment [50], so it is possible that adipocytes in the three groups of samples played a certain role in this study. Through transcriptome analysis, this study found that the up-regulated genes *EFHB* and *PER3*, as well as the down-regulated gene *PPARGC1A*, may be the main genes contributing to the differences in meat quality among the three groups.

Utilizing metabolomics to further investigate the differences in the impact of metabolites on meat quality among the three groups of sheep offered insights into the correlations between metabolite changes and the phenotypic characteristics of mutton. In this study, we found significant differences in metabolites such as L-histidinol, sarcosine, and gluconic acid among the different groups. Further KEGG analysis of the enrichment pathways of these important metabolites revealed that the DMs between the DH vs. HH groups were primarily enriched in metabolic pathways, thermogenesis, nucleotide metabolism, purine metabolism, and glycerophospholipid metabolism. The DMs between the SH vs. DH groups were primarily enriched in metabolic pathways, tyrosine metabolism, phenylalanine, tyrosine and tryptophan biosynthesis, carbon metabolism, nicotinate and nicotinamide metabolism, and butanoate metabolism. On the other hand, the DMs between the SH vs. HH groups were mainly enriched in metabolic pathways, purine metabolism, nucleotide metabolism, beta-alanine metabolism, taurine and hypotaurine metabolism, and biosynthesis of amino acids. Studies have shown that amino acids and peptide substances are some of the important prerequisite substances for meat flavor [51], L-histidinol is a precursor substance of histidine, and L -histidine and β-alanine synthesize myostatin with pH buffering, antioxidant, and carbonyl scavenging properties [52]. It has been reported that pork with a higher myostatin content in muscle has better quality, improved 24 pH, better tethering power, etc. [53]. Furthermore, L-histidine can also enhance the physicochemical properties of muscle proteins, such as solubility, gelation, and emulsification [54]. L-histidinol stands out as a crucial differential metabolite among the three groups, with significant up-regulation observed in both the DH vs. HH and SH vs. HH comparisons, indicating that the muscle’s water-holding capacity is superior in the DH vs. SH group compared to the HH group. It has also been shown that L-histidine affects the protein phosphorylation process of troponin C (TnC) through the activation of cAMP-dependent kinase (PKA) phosphorylation [55], and protein phosphorylation regulates metabolism, signaling, and key biological processes, such as proliferation and differentiation [56]. The metabolite of L-histidine, histamine, can enhance the expression of IGF-1 (Insulin-like Growth Factor-1) in ischemic muscle. Furthermore, as a cofactor acting on the histamine H3 receptor, histamine activates the PI3K/AKT signaling pathway within myoblasts, which plays a crucial role in muscle growth and tissue regeneration [57]. In the present study, a significantly different carbohydrate and its metabolite, gluconic acid, were also detected in the SH vs. HH group. Gluconic acid is formed by the microbial oxidation of glucose, which is an important substance in carbohydrate metabolism and an essential substrate for the de novo synthesis of fatty acids [58]. The differential metabolites L-histidinol and gluconic acid may have influenced the formation of phenotypic traits such as meat quality.

## 5. Conclusions

In this study, we conducted a detailed analysis and comparison of the muscle fiber diameter, cross-sectional area, and collagen fiber content of the *longest dorsal muscles* in crossbred sheep originating from various sire breeds. Additionally, we performed a comprehensive transcriptomic and metabolomic examination of these muscles. The results showed that the muscle fiber diameter, cross-sectional area, and collagen fiber content of the crossbred lambs were significantly increased compared with the Hu sheep. Through comprehensive transcriptome and metabolome analyses, we found that the *PER3* and *EFHB* genes may be related to muscle growth and development. Additionally, amino acid metabolism (especially histidine metabolism, arginine and proline metabolism, and phenylalanine metabolism) and carbohydrate metabolism play significant roles in regulating the meat quality of hybrid sheep. This study provides valuable information for a further understanding of the biological mechanisms underlying meat quality traits.

## Data Availability

The original data presented in the study are openly available in the NCBI Sequence Read Archive (SRA) database (PRJNA1213014).

## Supplementary Materials

The following supporting information can be downloaded at https://www.mdpi.com/article/10.3390/foods14081384/s1, Table S1: Composition and nutrient levels of basic diets (DM basis); Table S2: Details of primer sequences of miRNAs used for qRTPCR; Table S3: Statistics of RNA-Seq data quality; Table S4: Differentially expressed genes in longissimus dorsi; Table S5: GO enrichment analysis; Table S6: KEGG enrichment analysis; Table S7: All differential metabolites of longissimus dorsi; Table S8: Differential metabolites of longissimus dorsi; Table S9: KEGG pathway enrichment analysis of LC-MS; Table S10: Transcriptome and metabolic group KEGG were analyzed jointly; Table S11: Correlation statistics of differentially expressed genes and differentially expressed metabolites between metabolome and transcriptome.
