# Supplementary material for: Transcriptome–Metabolome Analysis Reveals That Crossbreeding Improves Meat Quality in Hu Sheep and Their F1-Generation Sheep
Source: Foods. 2025 Apr 17;14(8):1384. doi: 10.3390/foods14081384 (PMC12026837; doi:10.3390/foods14081384)
Supplement: Supplementary file 1 [file foods-14-01384-s001.zip › Table S1.pdf]

Supplementary Table S1 Composition and nutrient levels of basic diets (DM basis)

Ingredients	Conten(%)	Nutrient levels	Content(%)
Oat hay	6.08	DM	61.68
Leymus Chinensis	13.04	CP	13.19
Corn silage	13.53	ADF	12.75
Corn	32.78	NDF	23.60
Megalac[1)]	6.08	Fat	3.19
Lamb9303[2)]	28.49	Starch	23.01
Total	100.00	Ca	0.52
		P	0.29
		Nem/（MJ/kg）	1.33
		Neg/（MJ/kg）	0.86

Megalac. Rumen fatty acid calcium, Yihai Jiali, Tianjin. [2)]. Lamb9303: mainly composed of soybean oil, soybean meal, cottonseed meal, rapeseed meal, DDGS, calcium carbonate, calcium nitrogen phosphate, sodium chloride, a variety of trace elements, a variety of vitamins; Crude protein content ≥32.0%, ash≤21.0%, calcium 1.4%-5.0%, total phosphorus≥0.60%, crude fiber ≤20.0%, lysine ≥1.0%, sodium chloride 1.5-5.0%,All nutritional indexes in the table are measured values.
