# Supplementary material for: Transcriptome–Metabolome Analysis Reveals That Crossbreeding Improves Meat Quality in Hu Sheep and Their F1-Generation Sheep
Source: Foods. 2025 Apr 17;14(8):1384. doi: 10.3390/foods14081384 (PMC12026837; doi:10.3390/foods14081384)
Supplement: Supplementary file 1 [file foods-14-01384-s001.zip › Table S3.pdf]

Supplementary Table S3. Statistics of RNA-Seq data quality.

Sample	Raw Reads	Clean Reads	Clean Base(G)	Error Rate(%)	Q20(%)	Q30(%)	GC Content(%)
HH1	68712772	66670264	10	0.03	97.98	94.17	52.42
HH2	63628648	61789900	9.27	0.03	98.01	94.24	52.73
HH3	63700072	61545494	9.23	0.03	98.07	94.39	51.83
HH4	61582480	59671502	8.95	0.03	97.98	94.18	52.22
HH5	62263808	60403646	9.06	0.03	97.97	94.13	52.11
HH6	78064096	75670690	11.35	0.03	98.01	94.21	51.37
HH7	63014776	61248616	9.19	0.03	97.97	94.1	51.19
DH1	68739664	66670640	10	0.03	98.04	94.27	51.71
DH2	65280234	63397834	9.51	0.03	97.97	94.13	51.96
DH3	61901968	60150410	9.02	0.03	98.02	94.19	51.48
DH4	66788088	64778114	9.72	0.03	98.06	94.33	51.3
DH5	67805162	65780788	9.87	0.03	97.99	94.17	51.59
DH6	62471804	60583940	9.09	0.03	97.96	94.1	51.8
DH7	71342232	69083368	10.36	0.03	97.98	94.18	51.66
SH1	61732746	59728354	8.96	0.03	97.97	94.16	52.11
SH2	62880286	61064890	9.16	0.03	98.07	94.34	51.59
SH3	61178760	59505026	8.93	0.03	97.99	94.18	51.67
SH4	60618048	58853626	8.83	0.03	98.04	94.28	51.45
SH5	60615664	58827786	8.82	0.03	98.07	94.38	51.56
SH6	59919658	57916762	8.69	0.03	98	94.22	51.74
SH7	62174444	60368358	9.06	0.03	97.99	94.17	51.46

Error rate: overall sequencing error rate for the data; Q20 and Q30: percentages of total bases with Phred values above 20 and 30, respectively; GC pct: percentage of C and G among the 4 bases in clean reads.
